# Separation of simultaneously acquired [^89^Zr]atezolizumab and [^18^F]FDG PET scans

**DOI:** 10.1007/s00259-025-07340-w

**Published:** 2025-05-19

**Authors:** Zekai Li, Janneke W. de Boer, Tom van Meerten, Anne G. H. Niezink, Walter Noordzij, Adriaan A. Lammertsma, Charalampos Tsoumpas, Adrienne H. Brouwers

**Affiliations:** 1 Department of Nuclear Medicine and Molecular Imaging, University of Groningen, University Medical Center Groningen, Groningen, The Netherlands; 2 Department of Hematology, University of Groningen, University Medical Center Groningen, Groningen, The Netherlands; 3 Department of Radiation Oncology, University of Groningen, University Medical Center Groningen, Groningen, The Netherlands

[⁸⁹Zr]atezolizumab is a PET tracer that binds to programmed cell death ligand-1 (PD-L1), which is a key immune checkpoint expressed on a variety of human tumour types and immune cells and is an established target in cancer immunotherapy [1]. Across several tumour types, [⁸⁹Zr]atezolizumab PET uptake was better correlated to the response to PDL1-blockade therapy than immunohistochemistry or RNA-sequencing based PD-L1 expression levels [2]. [^18^F]FDG reflects glucose metabolism and is the most used PET tracer to stage patients with cancer. Traditionally, imaging with each tracer is performed on separate days. Simultaneous dual-tracer PET imaging offers the potential for integrated, complementary biological insights acquired under near-identical physiological conditions [3]. However, when two tracers are present in the patient, the overlapping signals make it impossible to distinguish their respective contributions to the overall signal. Although methodologies for separating dual-tracer signals have been explored for different tracer combinations [4, 5], clinical application has remained limited. The advent of long axial field-of-view (LAFOV) PET/CT systems enables whole-body imaging with enhanced sensitivity, creating new opportunities for simultaneous dual-tracer imaging. Here, we describe a patient with diffuse large B-cell lymphoma (DLBCL) who was scanned with both tracers on the same day using a LAFOV PET/CT system. We explore the feasibility of separating simultaneously acquired [⁸⁹Zr]atezolizumab and [^1^⁸F]FDG, using as prior knowledge the [^89^Zr]atezolizumab only scan performed earlier on the same day, thereby allowing to unveil distinct patterns unique to each tracer. The [^18^F]FDG scan was followed by a contrast enhanced CT covering the neck to pelvis confirming the FDG-avid lesions as compatible with sites of active lymphoma.

The patient underwent a 25 min one bed position PET/CT scan at 7 days post-injection (p.i.) of 37 MBq [⁸⁹Zr]atezolizumab. At the same day and 213 min after the first PET scan, 170 MBq [^18^F]FDG was administered, followed by a 5 min one bed position scan 65 min p.i. Both scans were performed using the Biograph Vision Quadra™ PET/CT scanner (Siemens Healthineers, Knoxville, TN, USA). Image reconstruction was performed using 3D ordered subset expectation maximization with 4 iterations, 5 subsets, and a matrix size of 220 × 220 × 645 (voxel size: 3.3 × 3.3 × 1.645 mm^3^), incorporating time-of-flight and point spread function modelling without post-reconstruction filtering.

To separate the tracer signals, a multi-step approach was implemented: CT-PET registration for each tracer [6], co-registration of PET datasets [7], and application of the extrapolation method for signal separation [8], which subtracts the extrapolated [⁸⁹Zr]atezolizumab signal from the dual tracer signal. After separation, an isotropic 5 mm full width at half-maximum Gaussian filter was applied. The maximum intensity projection images in standardized uptake value (SUV) (also shown in the animation, Online Resource 1) are displayed in two different ways: First, the [⁸⁹Zr]atezolizumab in red colour-scale, [^18^F]FDG in green colour-scale and their fusion which leads to a natural colour-scale reflecting not only the SUV of each tracer but also their presence/absence from each corresponding voxel. [^18^F]FDG images are presented using a red scale ranging from 0 to 7 SUV, while [⁸⁹Zr]trastuzumab images use a green scale ranging from 0 to 15 SUV. The fused dual-tracer images maintain the same respective SUV scales. A visually simpler display of the two tracers is depicted in the second row, in which [⁸⁹Zr]atezolizumab is shown in rainbow scale and [^18^F]FDG in standard grey-scale along with their simple fusion. The last colour scales could especially be more appreciated by readers who are colour-blind.

Separated [⁸⁹Zr]atezolizumab and [^18^F]FDG images revealed three distinct uptake patterns for lesions: (a) high uptake for both tracers (red arrow), (b) high [⁸⁹Zr]atezolizumab, but low [^18^F]FDG uptake (green arrow), and (c) high [^18^F]FDG, but low [⁸⁹Zr]atezolizumab uptake (blue arrow). The distinct uptake patterns observed for each tracer may provide valuable insights to further optimize and individualize patient treatment and patient selection. This study demonstrates the feasibility of separating simultaneously acquired [⁸⁹Zr]atezolizumab and [^18^F]FDG signals, using a same day [^89^Zr]atezolizumab only scan as prior knowledge. In the present protocol, ensuring accuracy and robustness of the image co-registration algorithm remains an important consideration. In theory, this can be circumvented by using protocols that enable simultaneous acquisition of [⁸⁹Zr]atezolizumab and [^18^F]FDG in a single PET imaging session, employing the same tracer separation methodology. Depending on the acquisition protocol used, simultaneous dual-tracer imaging would save the patient a visit to the hospital and/or reduce overall scanning time, thereby being more patient friendly and reducing scanning costs.
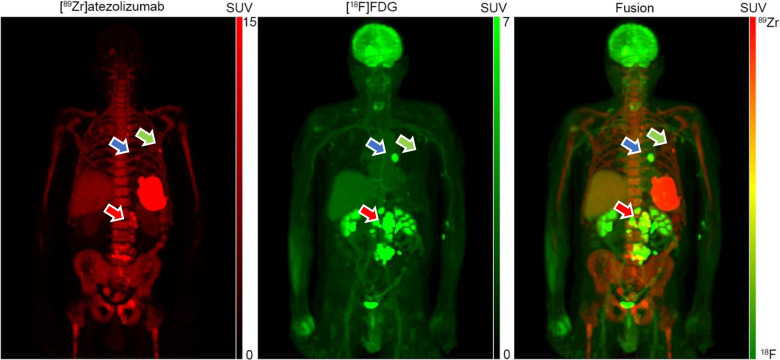



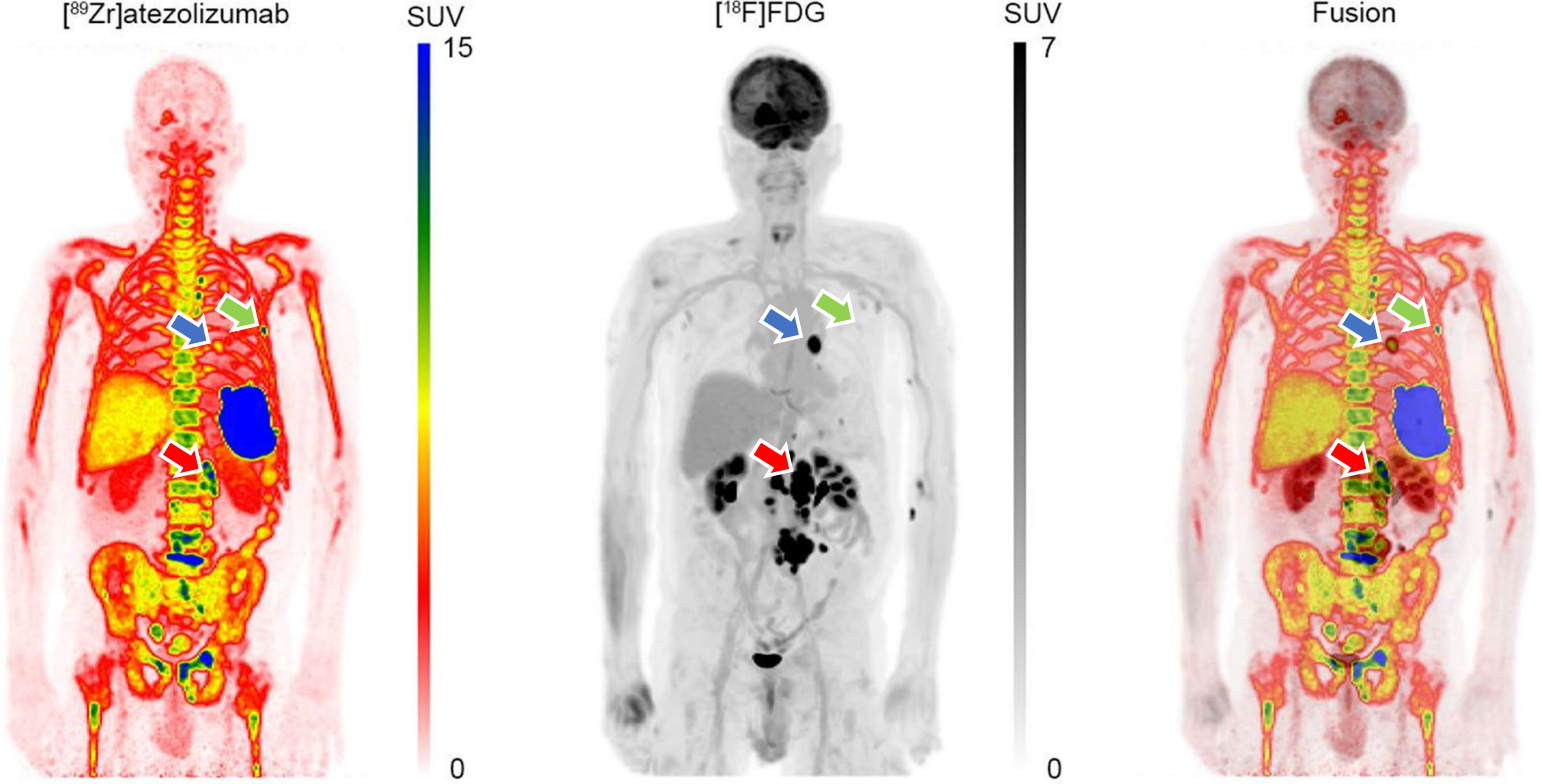


## Supplementary Information

Below is the link to the electronic supplementary material.

Supplementary file1 (GIF 9885 KB)

## Data Availability

No data will become available due to privacy concerns.
