# Supplementary figures and images for: Separation of simultaneously acquired [89Zr]atezolizumab and [18F]FDG PET scans
Source: Eur J Nucl Med Mol Imaging. 2025 May 19;53(2):705–7. doi: 10.1007/s00259-025-07340-w (PMC12830436; doi:10.1007/s00259-025-07340-w)

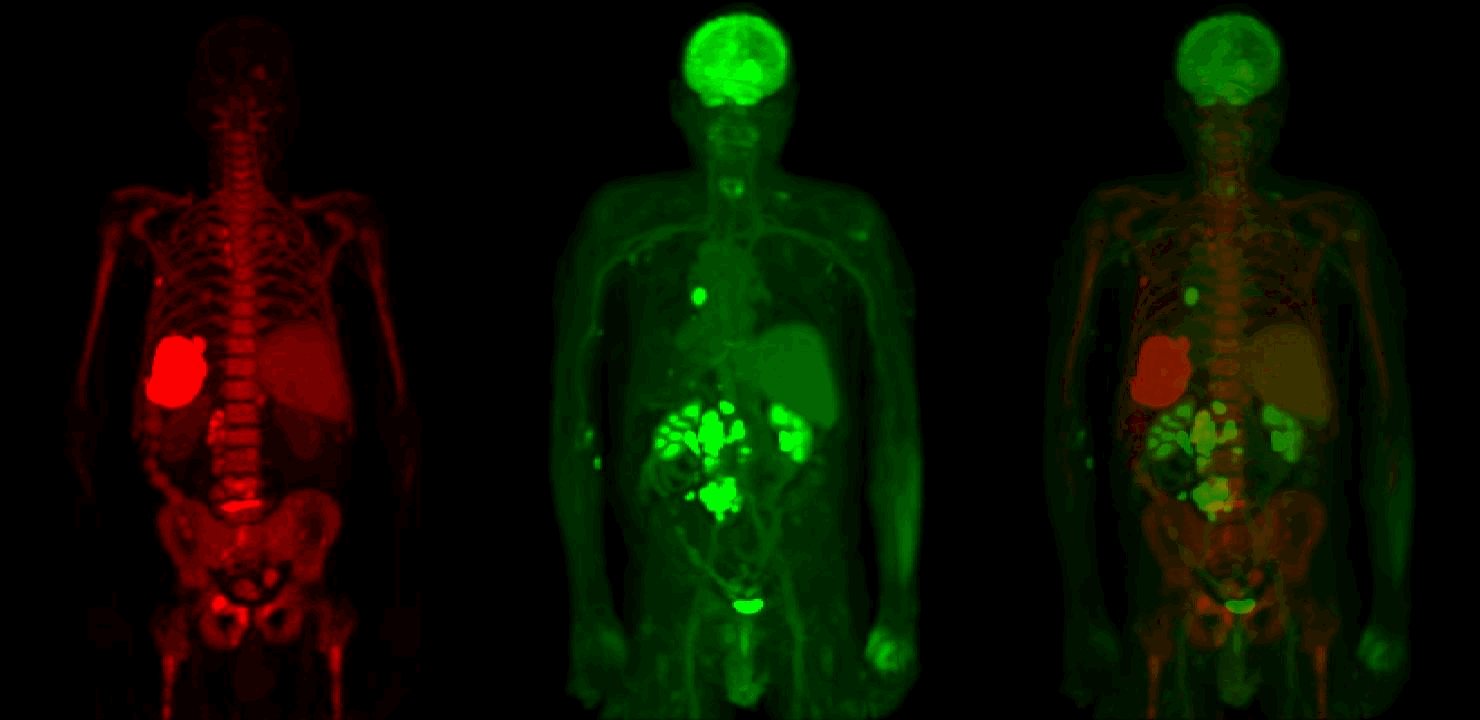

Supplement: Supplementary file 1 — Supplementary file1 (GIF 9885 KB) [file 259_2025_7340_MOESM1_ESM.gif]
